# Relationship between carotid atherosclerosis and cognitive impairment:A cross‐sectional study based on a population aged 40 years and older at high risk of stroke in a rural area of Xi'an

**DOI:** 10.1002/alz70860_098341

**Published:** 2025-12-23

**Authors:** Chen Chen, Ling Gao, Suhang Shang, Liangjun Dang, Shan Wei, Jingyi Wang, Jin Wang, Wenhui Lu, Qiumin Qu

**Affiliations:** ^1^ The First Affiliated Hospital of Xi'an Jiaotong University, Xi'an, Shaanxi, China; ^2^ The First Affiliated Hospital of Xi’an Jiaotong University, Xi'an, Shaanxi, China; ^3^ The First Affiliated Hospital of Xi'an Jiaotong University, Xi’an, China; ^4^ Huxian Hospital of Traditional Chinese Medicine, Xi'an, Shaanxi, China; ^5^ The First Affiliated Hospital of Xi'an Jiaotong University, Xi’an, Shaanxi, China

## Abstract

**Background:**

To explore the relationships between Carotid Atherosclerosis (CAS) and cognitive impairment in the stroke high‐risk population aged 40 years and above in the rural area of Xi 'an

**Method:**

In this study, stroke high‐risk population found in the “Community and rural Population Stroke High‐risk Group Screening and Intervention Project” carried out in HuYi District, Xi 'an City from October 2014 to March 2015 were selected as the research object. Color Doppler ultrasound was used to evaluate CAS, and CAS was defined as: (1) IMT≥1.0 mm; (2) Carotid arteries have atherosclerotic plaques, including common carotid artery, carotid sinus, internal carotid artery, and external carotid artery; (3) Carotid stenosis. Mini‐Mental State Examination (MMSE) was used to assess cognitive function, and the MMSE score was lower than the cut‐off value (illiteracy ≤17 points; Primary school ≤20 points; Junior high school and above education level ≤24 points) is defined as cognitive impairment. Univariate difference test and bivariate logistic regression were used to analyze the relationship between CAS and cognitive impairment.

**Result:**

A total of 451 subjects were included in the analysis. The average age of the subjects was 58.7±9.83 years old, and 44.3% were female. Among them, 329 cases (72.9%) had CAS and 57 cases (12.6%) met the diagnostic criteria of cognitive impairment. The prevalence of cognitive impairment in CAS group was significantly higher than that in non‐CAS group (14.6% vs.7.4%, *p* = 0.041). Multivariate logistic regression analysis showed that cognitive impairment was significantly correlated with stroke history (OR=11.924) and diabetes mellitus (OR=1.883), but not with CAS (OR=1.336, 95% CI 0.593‐3.010, *p* = 0.484).

**Conclusion:**

No significant association between CAS and cognitive impairment was found among high stroke risk group aged 40 and above in rural areas of Xi 'an.

